# Supplementary material for: Methylated Cell-Free DNA Sequencing (MeD-seq) of LpnPI Digested Fragments to Identify Early Progression in Metastatic Renal Cell Carcinoma Patients on Watchful Waiting
Source: Cancers (Basel). 2023 Feb 21;15(5):1374. doi: 10.3390/cancers15051374 (PMC10000042; doi:10.3390/cancers15051374)
Supplement: Supplementary file 1 [file cancers-15-01374-s001.zip › File S1.pdf]

Supplementary methods

Data analysis, calculation of RCC-specific methylation score

To identify differentially methylated regions (DMRs) between patients and HBDs in the dataset from Nuzzo et al. we used LIMMA[22]. A false discovery rate (FDR) was calculated to correct for multiple testing. Resulting DMRs were intersected with a composed list of 53 potential RCC methylation markers from literature [13-18], resulting in a panel of 22 validated RCC-specific cfDNA methylation markers (Figure 1).
To generate an overall methylation score per patient based on these 22 markers, we performed the following calculations using the normalized data per region as described before [20, 23], where i denotes HBD1,2,,,10, μ denotes average and σ denotes standard deviation.

1) For each HBDs a Z-score per region was calculated relative to the remaining 9 HBDs using a leave-one-out approach

$$HBD\ Z(region) = \frac{HBDi - \mu(HBD_{\neq i})}{\sigma(HBD_{\neq i})}$$

2) The obtained Z scores per region were squared and summed into 1 value per HBD

$$SUMSQ_{HBD} = \sum (HBD\ Z(region)_1, HBD\ Z(region)_2, \dots, HBD\ Z(region)_{22})^2$$

3) Z scores per region were calculated for every patient relative to the HBDs

$$Patient\ Z(region) = \frac{Patient - \mu(HBD)}{\sigma(HBD)}$$

4) The obtained Z scores per region were squared and summed into 1 value

$$SUMSQ_{patient} = \sum (Patient\ Z(region)_1, Patient\ Z(region)_2, \dots, Patient\ Z(region)_{22})^2$$

5) An RCC-specific summary score relative to HBDs was calculated

$$RCC\ summary\ score = \frac{SUMSQ_{patient} - \mu(SUMSQ_{HBD})}{\sigma(SUMSQ_{HBD})}$$
