# Supplementary figures and images for: Methylated Cell-Free DNA Sequencing (MeD-seq) of LpnPI Digested Fragments to Identify Early Progression in Metastatic Renal Cell Carcinoma Patients on Watchful Waiting
Source: Cancers (Basel). 2023 Feb 21;15(5):1374. doi: 10.3390/cancers15051374 (PMC10000042; doi:10.3390/cancers15051374)

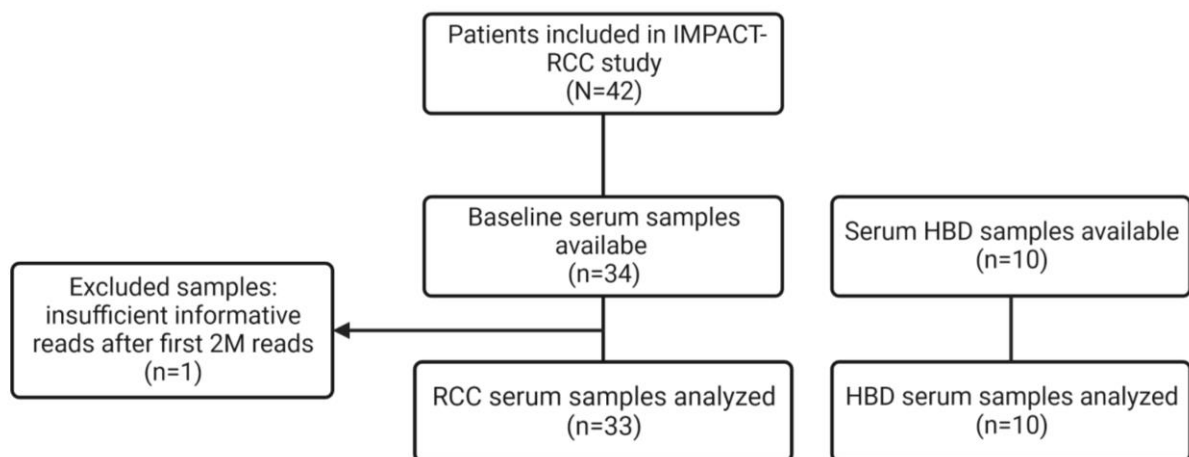

**Figure S1.** Flow chart of study samples

Supplement: Supplementary file 1 [file cancers-15-01374-s001.zip › Figure S1.pdf]
